# Correction of Length Discrepancy of Radius and Ulna with Distraction Osteogenesis: Three Cases

**DOI:** 10.1155/2015/656542

**Published:** 2015-08-10

**Authors:** Kenan Koca, Serkan Akpancar, Cemil Yıldız

**Affiliations:** Department of Orthopedic Surgery, Gulhane Military Medicine Academy, 06010 Ankara, Turkey

## Abstract

*Objectives*. The aim of the study was to investigate the results of patients with isolated length discrepancy between ulna and radius who underwent distraction osteogenesis with unilateral external fixator. *Material and Methods*. A patient with ulna shortening due to multiple enchondromatosis, a patient with ulna shortening due to ulnar club hand, and a patient with radial shortening due to radial club hand were included in the study. The patients underwent ulna and radial distraction osteogenesis with unilateral external fixator. Range of wrist and forearm motion, deformities, and length discrepancy of ulna and radius were compared at preoperative and postoperative. *Results*. Duration of external fixation and followup were 2.6 and 23.3 months, respectively. Mean distraction osteogenesis was 1.66 cm. No patient reached the length of normal side. Range of rotation of forearm was increased by 15°. Range of ulnar-radial deviation was increased by 21.6°. Deformity of 15° at patient with multiple enchondromatosis was corrected. *Conclusion*. Isolated ulna or radius shortening may reduce with distraction osteogenesis by unilateral external fixator to prevent serious deformity.

## 1. Introduction

Common causes of the radius and ulna length discrepancy in the childhood are radial club hand, ulnar club hand, traumatic epiphyseal injury, multiple osteochondromatosis, fibrocartilaginous focal dysplasia, and congenital pseudarthrosis [1]. Since the idea that advocated that upper extremity length discrepancy does not much affect the body functions and cosmetic appearance, upper extremity lengthening has not been as widespread as the lower extremities [2]. The unbalanced growth between the ulna and radius leads to deformity that significantly affects elbow, forearm, and wrist functions. In particular, the length difference will increase between the two bones in childhood which will also increase the deformity. So the prevention of unbalanced growth between the radius and ulna in forearm is thought to be necessary. In this study, we aimed to present the early clinical and radiological results of patients who underwent isolated ulnar or radial lengthening [3].

## 2. Material and Methods

Between September 2007 and December 2008 three patients were admitted to clinic with the complaint of isolated ulna and radius length discrepancy. One of the three patients had multiple enchondromatosis, the other had isolated ulnar shortening and deformity due to the ulnar club hand, and another had isolated radial shortening and deformity due to the radial club hand. With the aim of eliminating deformity and shortening, distraction osteogenesis was applied to shorter ulna or radius with osteotomies and unilateral external fixators.

### 2.1. Case 1

A seven-year-old male patient with multiple enchondromatosis was admitted to our clinic with complaints of left forearm and wrist deformity, length discrepancy between his upper limbs, and limited forearm rotation.

In the patient's physical examination, there was a significant length discrepancy between both upper extremities. Ulnar deviation of left forearm and wrist was observed. The supination and pronation of forearm and radial deviation of wrist were limited when compared to right extremity. Enchondromatosis lesions at the distal ulna were seen on radiographic evaluation. Ulna was short and deformed; distal ulna did not articulate with the distal radius and did not meet the carpal bones. Carpal bones replaced to ulnar side. The left ulna was found to be 2 cm shorter than right ulna.

To correct the deformity and shortening of ulna, osteotomy was performed to metaphyseal region of proximal ulna and unilateral external fixator was placed. After three days of operation, distraction osteogenesis was started and 1 mm (4 × 0.25) lengthening was performed daily. After the total of 2 cm distraction, lengthening was discontinued. Two months waiting for consolidation, external fixator was removed. During the distraction and consolidation the patient continued to use his hand in his daily activities and continued an exercise program to maintain the active and passive range of motion. There were no complications in this period, except for superficial infection at the bottom of the pin.

In the followup of the patient the elongation of the ulna had the corrective effect of the radius deformity. Restriction of radial deviation of the wrist and forearm rotation too much decreased. Two years later ulnar deformity and shortening recurred again and also radial head dislocation occurred in the patient. Ulnar osteotomy and distraction osteogenesis were applied again and elongated by 2 cm.

As a result of 3 years of followup the length of discrepancy between radius and ulna and deformity were significantly decreased. 1 cm length difference continued in ulna when compared with the healthy side. Preoperative, intraoperative, and postoperative clinical and radiographic images are shown in Figure 1.

### 2.2. Case 2

A 4-year-old female patient was admitted to clinic, because of deformity in the left wrist and forearm due to congenital anomalies. In the physical examination ulnar deviation in the wrist, the angulation in the forearm to ulnar side, and ulnar club hand were diagnosed. The patient had limitation of wrist radial deviation and forearm rotation. Wrist flexion extension was almost full of motion. The 5th finger was missing and had syndactyly between the second and the third fingers, as well as the third and fourth fingers. The patient was able to use this hand quite as active in daily activities.

On the radiographic evaluation significant shortening and mild angulation were present in the left ulna. Accordingly, significant angulation for compensation in the radius was detected.

In patient followup, the shortness of the ulna bone was further increased and consequently deformity of the ulna and radius deformity were increased. Ulnar deformity correction and lengthening surgery was planned. After the osteotomy that was applied to the proximal ulnar metaphyseal region, distraction osteogenesis with a unilateral external fixator was performed. 1 mm (4 × 0.25) lengthening was performed daily. After a total of 1,5 cm distraction, lengthening was discontinued due to excessive pain of the patient. External fixator was removed after waiting two months for consolidation. Then the orthosis that was used that forced to left wrist and forearm to radial deviation.

One year later the ulnar deviation of the wrist and angulation of the forearm were significantly decreased although an amount was still continuing. After 1.5 cm lengthening, distraction osteogenesis was stopped due to being unable to tolerate. Radial deviation in wrist was decreased but it was not corrected completely. There were no complications out of this. Preoperative, intraoperative, and postoperative clinical and radiographic images are shown in Figure 2.

### 2.3. Case 3

An 11-year-old male patient who received no treatment before was admitted to clinic due to the right forearm and wrist deformity. On the physical examination, radial deviation of the wrist and angulation of the forearm to the radial side were determined. Advanced limitations in ulnar deviation of the wrist and forearm rotation were observed in the patient. In the radiographic evaluation, radius was shorter and angulated to radial side but wrist was in a centralized position. As a compensatory measure, ulna was angulated to radial side. The patient was diagnosed with Heinke type 1 radial club hand. Deformity correction and distraction osteogenesis surgery was planned to the right radius. Osteotomy was performed to radius diaphyseal region and then distraction osteogenesis with unilateral external fixator was started. After a total of 1.5 cm, distraction was obtained and lengthening was discontinued. After 2 months of waiting for consolidation, external fixator was removed. During the distraction and consolidation, the patient was allowed to use hands in his daily activity and was given an exercise program to maintain the active and passive range of motion. No complications occurred to the patient.

Two years later despite having little shortness of the radius, the patient could easily move his wrist from neutral position to radial and ulnar deviation. Ulnar deviation of the wrist and forearm rotation was significantly increased. Preoperative, intraoperative, and postoperative clinical and radiographic images are shown in Figure 3.

## 3. Results

Average of 2.33 cm osteogenesis was applied to patients (1.5 cm in two patients, one patient 4 cm). The average duration of external fixation time was 3,4 months and total followup was 39.3 months (one patient 3 years, one patient 2 years, and one patient 1 year). The average elongation of bones is 2.3 cm. The forearm rotation increase average of 15 degrees and wrist ulnar-radial deviation were increased in an average of 21.6 degrees. None of the patients reached to the healthy side length (Tables 1 and 2). Shortness of ulna was elongated by less than planned due to the extreme amount of pain in the second patient. The deformity of 15° which is caused by multiple enchondromatosis was corrected in the first case.

## 4. Discussion

There are many causes of imbalanced growth in the forearm between the radius and ulna. Epiphyseal injury due to trauma, multiple osteochondromatosis, radial club hand, and ulnar club hand are some of those reasons. Especially in childhood, the unbalanced growth of the two forearm bones may lead to loss of function or severe deformities in forearm. The causes of unbalanced growth between the radius and the ulna that were included in the study are multiple enchondromatosis, ulnar club hand, and radial club hand. All of the patients had deformity on their forearm and had limitations of radial or ulnar deviation in their wrists and also limitations of internal-external rotation in their forearm.

According to the length discrepancy of the lower extremities, functional and cosmetic problems less frequently occur in the upper extremity. Therefore, surgical corrections of the upper limb length discrepancy are less frequently done compared to lower extremities [4]. However, unbalanced growth between the radius and ulna in the same forearm can cause deformity and loss of function at the wrist. In the case of isolated shortening of the radius and ulna, elimination of shortening is required to reduce the loss of function due to the increase of the deformity (Konrad Mader). In this study isolated radius and ulna shortening of the patients had led to severe deformity and loss of functions. Deformity of the limbs was found to increase with time.

Wagner first defined the grafting and fixation with plate-screw for the correction of the upper limb shortening and this method was used extensively in the past. It provides acute lengthening but the soft tissues do not often allow acute lengthening at the desired level [5]. Nowadays distraction osteogenesis with the Ilizarov circular external fixators is widely used in upper limb length discrepancy [6] and also lengthening of the isolated the radius or ulna shortening [7]. However, the circular fixator is more difficult to use in the upper limbs and there are disadvantages such as blocking muscle-tendon movement and causing neurovascular injury. Tetsworth et al. applied the Ilizarov circular external fixator to 19 upper extremities in order to have deformity correction or lengthening. They have encountered 9 of them major, a total of 14 complications [8]. Modern unilateral external fixator has developed in recent years for distraction osteogenesis and provided important advantages in the isolated ulna or radial shortening (Kondrad Mader). These advantages are easy usage of the fixator, low risk for neurovascular injury, and providing muscle-tendon movement. They are more comfortable for the patient [9]. On the other hand, the fracture that was occurred in the distraction site, after the external fixator is removed site is frequently reported complication in the literature [6]. Because of the advantages, we used unilateral external fixator for distraction osteogenesis to the isolated shortening of radius or ulna and encountered superficial pin tract infection in one patient and pain intolerance in the other patient.

The average age of the upper limb lengthening is still controversial in the literature. Some authors consider at early ages due to better remodelisation and to prevent progression of the deformity and some consider over 10 years and 0,5 cm–1 cm excess lengthening due to prevent the need of second surgical lengthening and deformity correction [4, 10]. We believe that distraction osteogenesis should be done at early stages in the patients whose deformity and loss of function can rapidly increase and over-lengthening method can reduce the need for second distraction osteogenesis. Therefore, distraction osteogenesis for the reduction of the deformity in the second case was performed in the age of 4 years. We did not prefer extreme lengthening due to the pain intolerance in our patients. However, the second-time distraction osteogenesis was performed to one patient due to growth and we think that there is a need to extend the second time in another patient.

The patients with nonfunctional hand and who have contractions in their elbow or wrists cannot be appropriate for lengthening. Moreover, lengthening could cause flexion contractures [11]. Thus detailed patient evaluation is as important as surgery.

There is limited literature available about deformity correction surgery in isolated radius and ulna lengthening due to the unbalanced growth in the same forearm. Villa et al. [6] reported that only one of the 12 patients had length discrepancy between the radius and ulna where they performed lengthening to the forearm. Huang and Kuo [3] reported 3 cases with length discrepancy, which was caused by multiple osteochondromatosis, fibrous dysplasia, and radial club hand. In one of these patients, deformity was corrected with isolated ulnar lengthening by Ilizarov external fixator, and the other two had implemented isolated radial lengthening surgery. The amount of elongation was between 21% and 50% of the bone. They did not report any significant complication. In our study we performed correction surgery of isolated length discrepancy for ulna and radius with the protocols that were similar to literature. Thus deformities reduced by decreasing the discrepancy in length and patient functions were improved.

As a result, deformity and loss of function due to the isolated radius or ulna shortening can be corrected with distraction osteogenesis method by unilateral external fixator without serious complications. However, deformity or shortening may recur after the first time of distraction osteogenesis and may need repetitive osteotomy and distraction osteogenesis surgery.

## Figures and Tables

**Figure 1:**
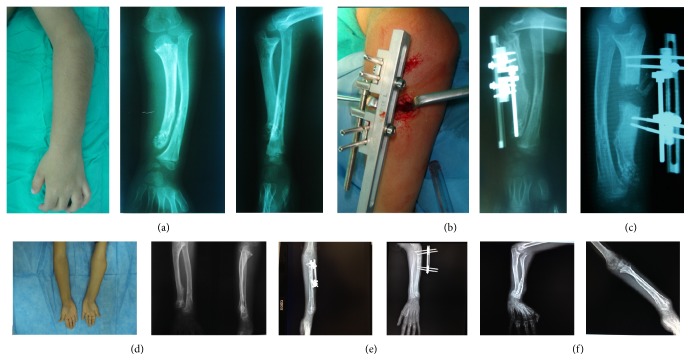
The preoperative, intraoperative, and postoperative images of the first case. (a) Preoperative and (b) intraoperative images of the first time of distraction, (c) 45 days of distraction osteogenesis, (d) clinical and radiographic images of patients after 2 years of distraction osteogenesis, (e) postoperative images of patients after second distraction osteogenesis, and (f) images of patients after implants were removed.

**Figure 2:**
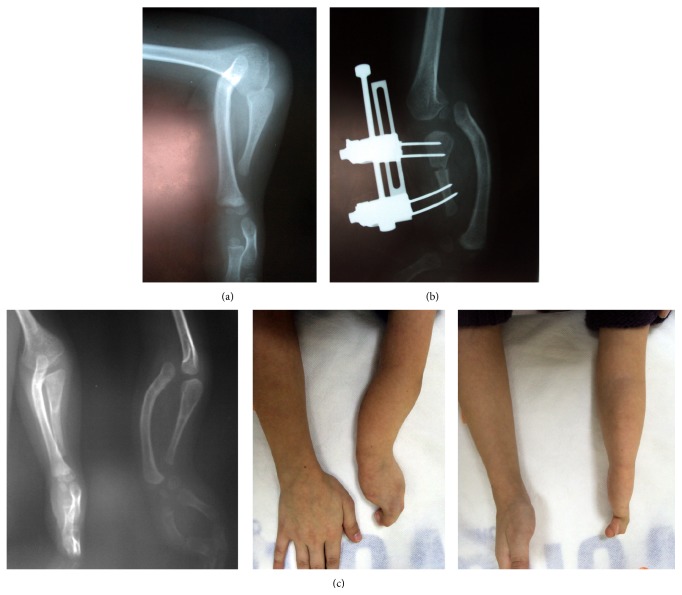
The preoperative, intraoperative, and postoperative images of the second case. (a) Preoperative and (b) intraoperative (c) images of patients after implants were removed.

**Figure 3:**
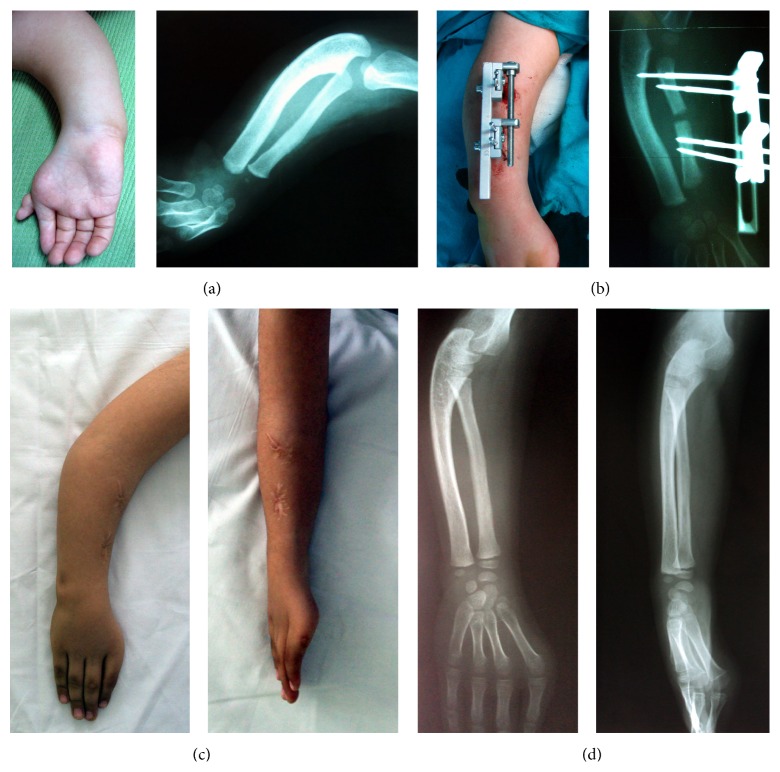
The preoperative, intraoperative, and postoperative images of the third case. (a) Preoperative and (b) intraoperative ((c), (d)) images of patients after implants were removed.

**Table 1:** Patient characteristics.

Patient	Age, sex	Side	Etiology	Lengthened bone	Followup time	Fixator time	Amount of lengthening (cm)	Complication
1	7, male	Left	ME	Ulna	24 months	14 weeks	2 + 2	Pin tract inf.
2	4, female	Left	UCH	Ulna	12 months	13 weeks	1.5	Pain intolerance
3	11, male	Left	RCH	Radius	24 months	11 weeks	1.5	None

AL: amount of lengthening, ME: multiple enchondromatosis, UCH: ulnar club hand, RCH: radial club hand.

**Table 2:** Preoperative and postoperative findings.

	Preoperative	Postoperative
Ulna length^#^	11 cm (1.op)-	14 cm (1.op)-
The contralateral length difference of ulna^#^	2 cm (1.op)-	1 cm (1.op)-
Ulnar deformity^#^	15°	0°
Range of motion of forearm rotation^#^	60°	80°
*Range* of *motion* of wrist radial deviation^#^	0°	20°
Ulna length^*μ*^	7	9
The contralateral length difference of ulna^*µ*^	3	2
Ulna deformity^*µ*^	0°	0°
Range of motion of forearm rotation^*µ*^	60°	75°
*Range* of *motion* of wrist radial deviation^*µ*^	−10°	15°
Radius length^*∗*^	16	18
The contralateral length difference of radius^*∗*^	3	2
Radius deformity^*∗*^	0°	0°
Range of motion of forearm rotation^*∗*^	40°	50°
Range of motion of wrist radial deviation^*∗*^	−20°	0°

^#^Ulnar lengthening performed in the first case.

^*μ*^Ulnar lengthening performed in the second case.

^*∗*^Radial lengthening performed in the third case.
